# Ability of the MeltPro MTB/PZA Assay to Detect Susceptibility to Pyrazinamide in Rifampin-Resistant Tuberculosis Patients

**DOI:** 10.1128/spectrum.04836-22

**Published:** 2023-05-10

**Authors:** Rong Li, Yang Li, Xinchang Chen, Lina Jia, Hongying Yu, Ya Huang, Qianhong Wu, Mingying Xiao, Shijia Ge, Yilin Zhang, Zhen Feng, Qingge Li, Ye Xu, Wenzhi Shi, Feng Sun, Wenhong Zhang

**Affiliations:** a Department of Infectious Diseases, Jing’an District Central Hospital, Fudan University, Shanghai, China; b Department of Infectious Diseases, Shanghai Key Laboratory of Infectious Diseases and Biosafety Emergency Response, National Medical Center for Infectious Diseases, Huashan Hospital, Fudan University, Shanghai, China; c Department of Tuberculosis, Guiyang Public Health Clinical Center, Guiyang, China; d Center for Infectious Diseases, The First People’s Hospital of Huaihua, Huaihua, Hunan, China; e Department of Tuberculosis, Ge Jiu Infectious Disease Hospital, Gejiu, China; f Department of Tuberculosis, Shaanxi Provincial Tuberculosis Prevention and Control Hospital, Xi’an, Shannxi, China; g Department of Tuberculosis, Baoshan People’s Hospital, Baoshan, China; h Engineering Research Centre of Molecular Diagnostics of the Ministry of Education, State Key Laboratory of Cellular Stress Biology, State Key Laboratory of Molecular Vaccinology and Molecular Diagnostics, School of Life Sciences, Faculty of Medicine and Life Sciences, Xiamen University, Xiamen, China; i National Clinical Research Center for Aging and Medicine, Huashan Hospital, Fudan University, Shanghai, China; j Key Laboratory of Medical Molecular Virology (MOE/MOH), Shanghai Medical College, Fudan University, Shanghai, China; k Shanghai Huashen Institute of Microbes and Infections, Shanghai, China; Johns Hopkins University School of Medicine

**Keywords:** pyrazinamide, melting curve analysis, MeltPro MTB/PZA assay, drug susceptibility testing

## Abstract

Prediction of susceptibility to pyrazinamide (PZA) directly from sputum has been challenging. The MeltPro MTB/PZA assay, based on melting curve analysis, can simultaneously detect Mycobacterium tuberculosis and the resistance to PZA from sputum. We aimed to evaluate the MeltPro MTB/PZA assay to predict PZA resistance among rifampin-resistant tuberculosis (RR-TB) patients. We prospectively enrolled RR-TB patients in the registered trials, and their baseline sputum samples were obtained to perform the assay and culture. DNA sequencing of culture isolates was analyzed and used as the reference standard. Sanger sequencing was performed for samples with discrepant results between next-generation sequencing (NGS) and the investigational assay. The main analysis was conducted in the population of patients with interpretable results by both NGS and the assay. A total of 239 patients with RR-TB were screened, and 220 underwent the MeltPro MTB/PZA assay. The assay provided no information for 25 of 220 patients (11.4%). Among the remaining 195 patients, 13 had negative culture or insufficient raw NGS sequencing data, and 15 had indeterminate assay results. A total of 167 patients were included in the main analysis. Against DNA sequencing, the sensitivity, specificity, and negative predictive value of the assay for detecting resistance to PZA were 91.4% (95% confidence interval [CI], 87.1% to 95.6%), 89.9% (95% CI, 85.3% to 94.5%), and 95.2% (95% CI, 91.9% to 98.4%), respectively. In conclusion, the MeltPro MTB/PZA assay is a fast semiautomatic molecular platform to rapidly predict resistance to PZA from sputum and holds promise as a screening tool with satisfactory sensitivity.

**IMPORTANCE** This study evaluated the accuracy of the MeltPro MTB/PZA assay at detecting the presence of PZA resistance through registered clinical trials. Compared to DNA sequencing, the assay had high sensitivity and negative predictive value, suggesting its potential utility as a screening tool in clinical practice. The assay could serve as an ideal primary screening tool in low PZA-resistant M. tuberculosis prevalence settings and could be used as an additional test to identify PZA resistance rapidly and initially in the RR-TB population.

## INTRODUCTION

Drug-resistant tuberculosis (DR-TB) continues to be a public health problem, taking a heavy toll on patients, communities, and health care systems (1). Pyrazinamide (PZA), a niacinamide analog (2, 3), plays a crucial role in TB chemotherapy, shortening the treatment period of TB from 9 to 12 months to 6 months by killing semidormant Mycobacterium tuberculosis. However, PZA resistance is prevalent among multidrug/rifampin-resistant TB (MDR/RR-TB) patients, with incidence ranging from 38% to 60.5% (4). Thus, for patients diagnosed with MDR/RR-TB, it is important to detect the presence of PZA resistance, which is significantly associated with longer treatment duration and worse outcomes (5).

The lack of approved rapid testing for PZA resistance and time-consuming phenotypic drug susceptibility testing (DST) delay the initiation of precision therapy to include or replace PZA by weeks (1). In addition, phenotypic PZA resistance *in vitro* is unreliable because of the acid pH requirement for drug activity; moreover, the most common problem of DST is false resistance, as a large inoculum of a susceptible strain can lead to false resistant results (6). Next-generation sequencing (NGS) is a promising detection tool, with a sensitivity of 91.3% and a specificity of 96.8% for diagnosing PZA resistance (7). Due to the need for professional knowledge and instruments, NGS may only be carried out in well-developed regions. Molecular tools for the rapid detection of rifampin resistance can help clinicians tailor regimes and make decisions (8, 9), while no rapid molecular detection method for PZA resistance is available in clinical settings, a situation which needs to be further updated and developed.

Molecular assays were based on the detection of mutations in the promoter and open reading frame regions of the *pncA* gene, encoding pyrazinamidase (10–12). As reported, resistance-related *pncA* mutations are scattered throughout the gene without hot spots, hindering the development of rapid and straightforward test assays, which tend to be based on probe detection or Sanger sequencing. Previous studies have reported a small number of novel methods to detect resistance to PZA from sputum, but the process was complicated and time-consuming in practice, taking up to 2 days to obtain results (13–18).

The MeltPro MTB/PZA test assay, developed by Zeesan Biotech (Xiamen, China), is an innovative molecular test to detect PZA resistance. It can be performed directly on sputum samples and has a short turnaround time and semiautomatic operation. The study aimed to evaluate the diagnostic accuracy of the investigational assay.

## RESULTS

### Study population.

**FIG 1:**
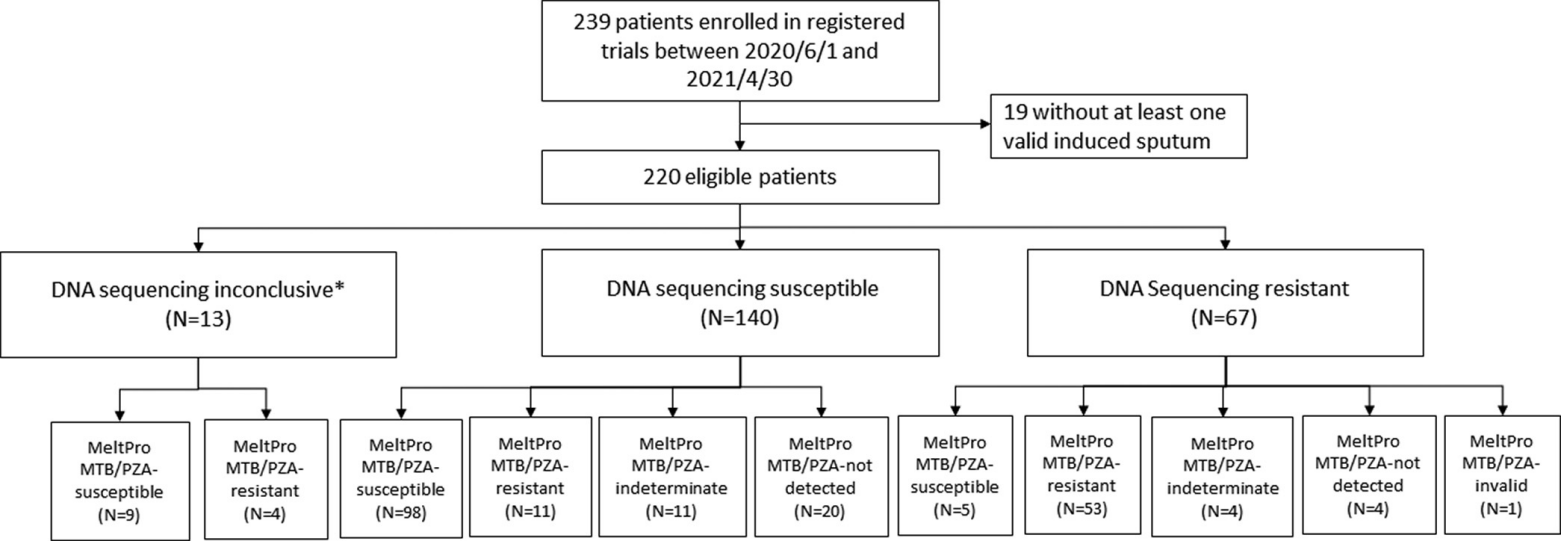
Patient enrollment and the MeltPro MTB/PZA assay results. *DNA sequencing results were inconclusive for 13 patients, including 12 with negative culture and 1 without sufficient raw NGS sequencing data.

From 1 June 2020 to 30 April 2021, a total of 239 patients with RR-TB were enrolled in the registered trials and screened for this study. After excluding 19 patients without sufficient sputum, the remaining 220 patients were eligible for this study, and testing was performed with the MeltPro MTB/PZA assay (Fig. 1). The demographic characteristics of the participants are shown in Table 1. Most participants (201/207; 97.1%) had a positive sputum smear, and 175 had cavitation. DNA sequencing identified 66 of 207 (31.9%) patients as having infections that were resistant to PZA (Fig. 1).

**TABLE 1:** Demographic and clinical characteristics of patients at enrollment^*a*^

Characteristic	No. of patients (%)	No. of patients (%) with:		*P* value
		Interpretable MeltPro MTB/PZA results	Uninterpretable MeltPro MTB/PZA results	
Total no. of patients	220 (100)	180 (100)	40 (100)	
Demographic characteristics				
Male sex	153 (69.5)	128 (71.1)	25 (62.5)	0.28
Age (yrs [mean ± SD])	41.7 ± 13.6	41.2 ± 13.3	44.0 ± 14.5	0.24
BMI (kg/m^2^ [mean ± SD])	20.2 ± 2.9	20.1 ± 2.9	20.5 ± 3.2	0.41
Previous treatment for TB	162 (73.6)	137 (76.1)	25 (62.5)	0.08
Clinical characteristics				
Cough	121 (55.0)	101 (56.1)	20 (50.0)	0.48
Expectoration	100 (45.5)	81 (45.0)	19 (47.5)	0.77
Hemoptysis	31 (14.1)	27 (15.0)	4 (10.0)	0.41
Fever	8 (3.6)	7 (3.9)	1 (2.5)	1.00
Night sweats	9 (4.1)	9 (5.0)	0 (0.0)	0.32
Wt loss	36 (16.4)	32 (17.8)	4 (10.0)	0.23
Sputum smear result				
Negative	8 (3.6)	7 (3.9)	1 (2.5)	0.14
Scanty	25 (11.4)	18 (10.0)	7 (17.5)	
Smear positive				
+	73 (33.2)	56 (31.1)	17 (42.5)	
++	52 (23.6)	44 (24.4)	8 (20.0)	
+++	33 (15.0)	27 (15.0)	6 (15.0)	
++++	29 (13.2)	28 (15.6)	1 (2.5)	
Radiographic findings				
Bilateral involvement	178 (80.9)	147 (81.7)	31 (77.5)	0.54
Presence of cavity	188 (85.5)	162 (90.0)	26 (65.0)	<0.01

a N, number; PZA, pyrazinamide; BMI, body mass index; TB, tuberculosis.

### Operational characteristics of the MeltPro MTB/PZA assay.

Using the MeltPro MTB/PZA assay, a total of 40 patients (18.2%; 40/220) had no interpretable results. The MeltPro MTB/PZA assay provided no information for 25 of these patients (220 total patients; 11.4%), including 24 for whom the assay reported that M. tuberculosis was not detected and 1 with an invalid result. The MeltPro MTB/PZA assay sensitivity for M. tuberculosis detection was 88.2% (95% confidence interval [CI], 83.9% to 92.4%). Furthermore, 15 (6.8%) patients had indeterminate results with the MeltPro MTB/PZA assay. The proportion of cavity presence was significantly lower among patients without interpretable results than among those with interpretable results (65.0% versus 90.0%, *P* < 0.01) (Table 1).

The median time to detection of tuberculosis for the assay was 4 h (interquartile range, 3.5 to 4.5 h), which included specimen digestion and decontamination, the PCR protocol, and melting curve analysis and reporting.

### Performance of the MeltPro MTB/PZA assay for detection of resistance to PZA.

Of 180 patients with interpretable results with the MeltPro MTB/PZA assay, 13 had negative culture or insufficient raw NGS sequencing data. Therefore, a total of 167 patients were included in the main analysis. The sensitivity, specificity, positive predictive value, negative predictive value, and accuracy of the MeltPro MTB/PZA assay for detecting resistance to PZA were 91.4% (95% CI, 87.1% to 95.6%), 89.9% (95% CI, 85.3% to 94.5%), 82.8% (95% CI, 77.1% to 88.5%), 95.2% (95% CI, 91.9% to 98.4%), and 90.4% (95% CI, 86.0% to 94.9%), respectively. The diagnostic performance of the investigational assay was slightly improved in patients with lung cavities, for whom the sensitivity, specificity, positive predictive value, negative predictive value, and accuracy of the MeltPro MTB/PZA assay were 94.5% (95% CI, 90.9% to 98.2%), 94.7% (95% CI, 91.1% to 98.3%), 91.2% (95% CI, 86.7% to 95.8%), 96.7% (95% CI, 93.9% to 99.6%), and 94.6% (95% CI, 91.0% to 98.3%), respectively (Table 2).

**TABLE 2:** Performance of the MeltPro MTB/PZA assay for PZA resistance^*a*^

Study population	No. of:					% (95% CI)				
	Patients	TP	FP	FN	TN	Sensitivity	Specificity	PPV	NPV	Accuracy
Total	167	53	11	5	98	91.4 (87.1–95.6)	89.9 (85.3–94.5)	82.8 (77.1–88.5)	95.2 (91.9–98.4)	90.4 (86.0–94.9)
Patients with lung cavity	149	52	5	3	89	94.5 (90.9–98.2)	94.7 (91.1–98.3)	91.2 (86.7–95.8)	96.7 (93.9–99.6)	94.6 (91.0–98.3)

a TP, true positives; FP, false positives; FN, false negatives; TN, true negatives; CI, confidence interval; PPV, positive predictive value; NPV, negative predictive value; PZA, pyrazinamide.

### Discrepant results between the MeltPro MTB/PZA assay and NGS.

The assay correctly identified the mutant population in 53 of 58 (91.4%) sequencing-confirmed resistant populations. Isolates with inconsistent results between NGS and the assay were sent for Sanger sequencing, as shown in Table 3. Five and eleven samples were misdiagnosed as susceptible or resistant, respectively, by the MeltPro MTB/PZA assay.

**TABLE 3:** Discordance between MeltPro MTB/PZA and NGS results^*a*^

Isolate	MeltPro MTB/PZA mutation	NGS mutation	Sanger sequencing mutation
MeltPro MTB/PZA-S NGS-R			
42	WT	*pncA*_W119R	355T→C
208	WT	*pncA*_A146T	436G→A
P-057	WT	*pncA*_I6T	17T→C
231	WT	*pncA*_I6T	17T→C
R-027	WT	*pncA*_47GG_deletion	46–47 deletion
336	WT	*pncA*_Q10 (10%)^*b*^	WT
R-019	WT	*pncA*_D63A (29%)^*b*^	WT
MeltPro MTB/PZA-R NGS-S			
18	Missing *pncA* fragments	WT	Failed
72	Missing *pncA* fragments	WT	Failed
247	Missing *pncA* fragments	WT	Failed
P-020	Missing *pncA* fragments	WT	Failed
141	Missing *pncA* fragments	WT	WT
32	Missing *pncA* fragments	WT	WT
139	10–38	WT	WT
136	200–234	WT	WT
R-004	277–312	WT	WT
79	300–335	WT	WT
359	543–581	WT	WT
P-083	185–216	WT	196–206 deletion
143	Missing *pncA* fragments	WT	399–408 deletion
4	185–218	WT	638a insertion

a After the samples were sent for Sanger sequencing, the assay results were still inconsistent with Sanger sequencing or Sanger sequencing failed. If results with Sanger sequencing failed, the NGS results were taken as the reference method. Abbreviations: PZA, pyrazinamide; NGS, next-generation sequencing; R, resistant; S, susceptible; WT, wild type.

b Possibly due to heterogeneity in isolates.

In total, 7 isolates were susceptible according to the MeltPro MTB/PZA assay but resistant according to NGS; 5 of these were confirmed by Sanger sequencing to possess the same mutation detected by NGS. For the other 2 isolates (336 and R-019) confirmed to be susceptible by Sanger sequencing, the rates of *pncA* mutation detected by NGS were 10% and 29%, respectively, indicating heterogeneity of the isolates (Table 3).

Initially, a total of 14 NGS-susceptible strains were misdiagnosed as resistant by MeltPro MTB/PZA, of which 7 isolates were confirmed as *pncA* wild type by Sanger sequencing. Four out of fourteen MeltPro MTB/PZA-resistant and NGS-susceptible isolates failed using Sanger sequencing, and NGS was still taken as the gold standard. Of 3 isolates confirmed to be resistant by Sanger sequencing, P-083 (deletion in positions 196 to 206) and 143 (deletion in positions 399 to 408) were missed by NGS due to the limitations of our routine analysis pipeline, which omits deletions or insertions of more than 7 bp (Table 3).

NGS results were not obtained for 12 patients with culture-negative samples and 1 with insufficient raw data for NGS analysis. The assay detected PZA resistance in 4 patients and PZA susceptibility in 9 patients (Fig. 1).

### Indeterminate results with the MeltPro MTB/PZA assay.

In the first round of testing, 18 results were indeterminate, 33 were M. tuberculosis not detected, and 1 was invalid. Nineteen patients with sufficient sputum left were retested with the MeltPro MTB/PZA assay; 13 of them had explicable results, and the rest remained inconclusive (see Fig. S1 in the supplemental material). The sensitivity and specificity of the assay were 85.5% (95% CI, 76.7% to 94.3%) and 81.7% (95% CI, 74.7% to 88.6%), respectively, when the indeterminate results were included.

## DISCUSSION

This study reports the diagnostic accuracy of a rapid, semiautomated MeltPro MTB/PZA assay to detect the *pncA* gene and *pncA* promoter region in M. tuberculosis directly from sputum specimens, for the first time. Compared to DNA sequencing, the assay demonstrated the potential to detect PZA susceptibility with high sensitivity and a negative predictive value of greater than 90%, suggesting its potential utility as a screening tool. As the negative predictive value would further decline with an increasing prevalence of resistance to PZA, we can imagine that the MeltPro MTB/PZA assay will yield better diagnostic performance as a screening tool among populations with a lower prevalence of resistance to PZA, such as those infected with drug-susceptible TB.

Recently, the WHO has recommended rapid, culture-free, PCR-based tests, such as the Xpert MTB/RIF assay for rifampin, to identify bacteria in sputum to the species level and detect common drug resistance-related mutations (19). A dozen studies have focused on rapid molecular detection platforms for PZA directly from sputum, most of which were based on the line probe assay, colorimetric methods, targeted deep sequencing, whole-genome sequencing, or *pncA* sequencing (11, 13–18, 20–22). In studies by Mitarai et al. and Driesen et al., the approaches had disadvantages such as complex operation and complicated interpretation of results, while the MeltPro MTB/PZA assay provides a fully automated process, from PCR to resistance results (13, 22). In studies by Li et al. and Tam et al., the approaches based on *pncA* sequencing took up to 2 to 4 days to report PZA susceptibility, but according to the operating manual, the assay only took 4 h, and the operating time for the assay was under half an hour (14, 16). For other studies using targeted deep sequencing or whole-genome sequencing, the detection techniques required professional operation for DNA extraction, library construction, and analysis of the whole-genome sequencing data afterward; such techniques cannot be deployed in underresourced areas or primary hospitals. The sensitivity of these techniques for detecting resistance to PZA from clinical sputum samples ranged from 62% to 74%, which was lower than that in this study (15, 17, 18).

Mutations in the *pncA* gene represent the major PZA resistance mechanism and are found in >90% of PZA-resistant isolates (23). Only mutations associated with the *pncA* gene, other than known structural genes (*rpsA*, *panD*), were detected in our study, which may be related to gene differences among isolates from different regions (24). This indicates that the MeltPro MTB/PZA assay is likely to be the most suitable screening tool for the prediction of PZA resistance because it interrogates the *pncA* gene, which has higher mutation frequency, and its promoter regions. The prevalence of PZA resistance was 67/207 (32.4%) in this study, which is comparable with the national level (25). We believe that our sample is representative of patients with MDR/RR-TB and that our results can be extrapolated to a general MDR/RR-TB population.

On initial testing of the 220 specimens with the MeltPro MTB/PZA assay, inconclusive readouts (invalid, M. tuberculosis not detected) were obtained for 34 (15.5%) specimens. Upon retesting, inconclusive readouts were obtained for 9 (4.1%) specimens with the assay. The assay detected M. tuberculosis in 12 patients who were culture negative; M. tuberculosis was also confirmed using the Xpert MTB/RIF assay. The MeltPro MTB/PZA assay did not perform well in detecting M. tuberculosis in patients without cavities or those with lower smear grades, suggesting that further investigation is urgently needed to improve the results for patients with a low bacillary burden. It is necessary for us to evaluate the ability of this assay to screen for M. tuberculosis in people with confirmed active TB. Future contact tracing studies combining bacterial load and the MeltPro MTB/PZA should address this issue prospectively.

Despite being simple and rapid to use, the assay has several limitations. First, the phenotypic DST for PZA was not performed in this study, which leaves some doubt about the diagnostic performance of the MeltPro MTB/PZA assay compared to that of the gold standard test. Second, the performance of the assay was evaluated in mostly smear-positive patients. Considering the poor performance of the assay with samples from patients without cavities or those with lower smear grades, the results of this study need to be confirmed in more smear-negative patients. Finally, the discordance in results between NGS and the MeltPro MTB/PZA assay due to variation in the sample types also needs to be tested in future research.

PZA is a crucial component of the most commonly used short-course regimen for the treatment of TB. Therefore, it is necessary to identify resistance to PZA. In areas with low prevalence of PZA resistance, the MeltPro MTB/PZA assay could serve as an ideal primary screening tool. In the RR-TB population, it may be used as an additional test to rapidly and initially identify PZA resistance, and NGS can be used for subsequent verification.

## MATERIALS AND METHODS

### Study participants and procedures.

We enrolled participants in this study from three registered trials in China (NCT03867136, NCT04717908, NCT05081401) between 1 June 2020 and 30 April 2021 (26). The registered trials were optimized regimens for DR-TB defined by the rapid automated molecular assay. The study enrolled patients in the trials who were able to provide enough sputum samples after completing the rapid molecular assay. If the patients could produce extra sputum of more than 0.5 mL, the sputum would be tested using the investigational assay. Baseline sputum culture was performed for all patients in the registered trials, and positive cultures were sent for NGS.

In addition, Sanger sequencing was used in all discordant results. If the results of NGS and Sanger sequencing were inconsistent, the Sanger sequencing results were taken as the gold standard. If the results with Sanger sequencing failed, the NGS results were taken as the reference method. Demographic information, medical history, and chest imaging results were recorded at enrollment. Samples were obtained under informed consent with approval by the Ethical Committee of Huashan Hospital.

### MeltPro MTB/PZA assay.

DNA was extracted from the sputum using an automated DNA extraction workflow (LabAid-824S; Zeesan Biotech, Xiamen, China) within half an hour of lysing the sputum with the buffer included in the LabAid-824S (27). The MeltPro MTB/PZA assay was then performed following the manufacturer’s instructions. Briefly, the assay employed PCR and melting curve analysis methods in 4 separate tubes, covering mutations in *pncA* (561 bp) and the *pncA* promoter region (approximate position, −16 to −1) to determine the resistance to PZA, providing a fully automated process. Positive results for the assay were reported as M. tuberculosis positive, and the resistance target was resistant, susceptible, or indeterminate. An explanation of the assay results is provided in the supplemental material.

### Next-generation sequencing.

As the reference standard in the study, NGS was performed on all culture-positive strains independently to predict genotypic resistance. All colonies were scraped from the culture medium to maintain the diversity. Genomic DNA was extracted using the total nucleic acid extraction assay (IngeniGen XMK Biotechnologies, Inc., Zhejiang, China), and sequencing libraries were constructed using the DNA library preparation assay (IngeniGen XMK Biotechnology) following the manufacturer’s protocol. All strains were sequenced on the NovaSeq 6000 system with at least 100-fold coverage.

NGS data were analyzed according to previous studies (28–30). All single nucleotide polymorphisms (SNPs) with a mutation frequency exceeding 10% were retained during the step that involved calling SNPs to detect minor subpopulations. Due to the limitations of NGS (i.e., its inability to detect large fragment deletions), Sanger sequencing was performed and used as the final diagnostic result for all strains with incongruent MeltPro MTB/PZA assay and NGS results. Isolates possessing nonsynonymous mutations or frameshift mutations in *pncA* were diagnosed as resistant to PZA by NGS.

### Statistical analysis.

The sensitivity, specificity, positive predictive value, negative predictive value, and accuracy of the assay in detecting PZA resistance were calculated. When test results were classified as M. tuberculosis negative, invalid, or indeterminate, the tests were repeated as long as sufficient sputum samples were available. The repeated test results were used for analysis. When the results of the second test were still inconclusive (M. tuberculosis negative, invalid, or indeterminate), the results of the assay were listed. Categorical variables were compared using the chi-square test, and continuous variables were compared using the independent-sample *t* test. A *P* value of <0.05 was considered statistically significant. All statistical tests were performed using Stata 16.0 software (Stata Corporation, College Station, TX).
