# Supplementary material for: Ability of the MeltPro MTB/PZA Assay to Detect Susceptibility to Pyrazinamide in Rifampin-Resistant Tuberculosis Patients
Source: Microbiol Spectr. 2023 May 10;11(3):e04836-22. doi: 10.1128/spectrum.04836-22 (PMC10269598; doi:10.1128/spectrum.04836-22)
Supplement: Supplemental file 1 — Supplemental material. Download spectrum.04836-22-s0001.pdf, PDF file, 0.5 MB [file spectrum.04836-22-s0001.pdf]

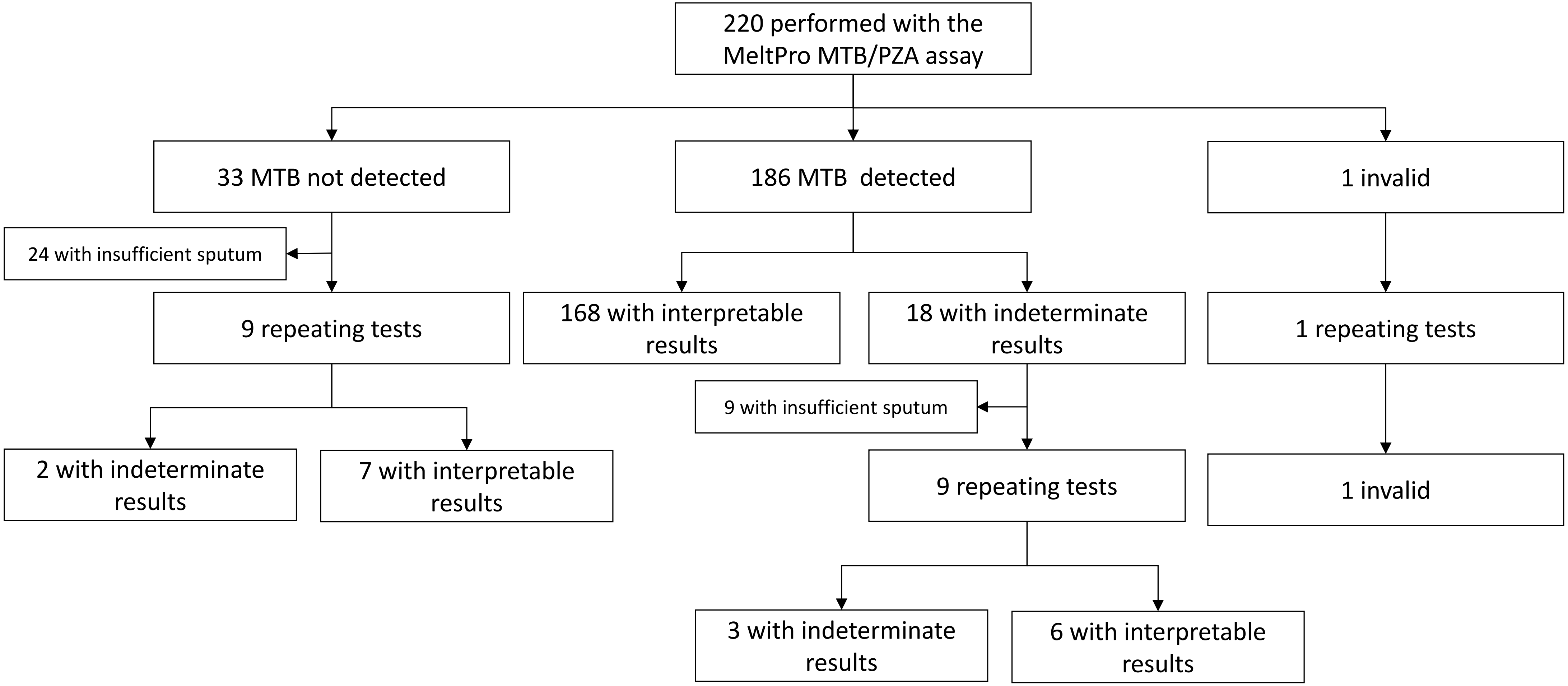

Figure S1 The MeltPro MTB/PZA test results for all 220 patients and repeat test results classified as "MTB not detected", "invalid" or "indeterminate"

Abbreviation: MTB, *Mycobacterium tuberculosis*; PZA, pyrazinamide

INTERPRETATION OF THE RESULTS

I. REFERENCE VALUE

The controls in the kit must meet the following requirements, otherwise, the experiment will be considered invalid. The *Tm* value range of positive control (wild type) in each channel of A, B, and C reaction is shown as follows:

Reaction System	Channel	*Tm* value(°C)		
		*Tm*1	*Tm*2	*Tm*3
A	FAM	46.1±1.6(1)	58.5±1.6(3)	69.2±1.6(8)
	HEX	46.0±1.6(14)	58.3±1.6(17)	68.5±1.6(34)
	ROX	52.7±1.6(24)	61.8±1.6(12)	68.5±1.6(34)
	Cy5	50.2±1.6(26)	61.6±1.6(21)	70.6±1.6(5)
B	FAM	53.3±1.6(11)	60.5±1.6(2)	67.7±1.6(36)
	HEX	48.2±1.6(27)	58.2±1.6(33)	67.2±1.6(31)
	ROX	55.0±1.6(18)	63.2±1.6(6)	70.8±1.6(29)
	Cy5	51.0±1.6(15)	60.7±1.6(9)	68.6±1.6(22)
C	FAM	45.7±1.6(10)	58.9±1.6(16)	68.9±1.6(23)
	HEX	50.1±1.6(35)	62.4±1.6(7)	73.3±1.6(25)
	ROX	45.6±1.6(20)	59.3±1.6(28)	65.5±1.6(30)
	Cy5	50.7±1.6(13)	59.2±1.6(4)	69.0±1.6(19)

Note: The sequence numbers of the melting peaks are in parentheses, from 1 to 36 in order.

Reaction D only gives amplification signals. For positive control and negative control, their Ct values range are as follows:

Positive control: FAM channel: Ct value is less than 18.5 (Ct<18.5);
HEX channel: Ct value is less than 27.0 (Ct<27.0).

Negative control: FAM channel: No Ct;
HEX channel: Ct value is less than 27.0 (Ct<27.0).

Always calibrate the above *Tm* values with the positive control (wild-type) for each run. The *Tm* values will be given automatically by the instrument.

II. EXPLANATION OF RESULTS

1. **Interpretation of positive control:** For each run, the *Tm* value of positive control of all channels should be within the range of reference values, otherwise the experiment will be considered invalid.

2. **Interpretation of negative control:** There should be no signal observed for negative control in each channel except D-HEX, which indicates that there is no contamination during DNA extraction or detection. Otherwise the experiment will be considered invalid.

3. **Interpretation of specimens:**

1) Whether a specimen is a mutant or not is determined by comparing the difference in *Tm* values between the specimen and the positive control:

-△*Tm*= *Tm* (positive control) - *Tm*(specimen)

- Wild peak: -1.6°C < △*Tm* < 1.6°C

- Mutant peak: |△*Tm*|≥1.6°C

The result is valid when the △*Tm*≥1.6°C of the third melting peak (△*Tm*3) in any channel, it is suggested to check the sample quality; or repeat test is recommended to confirm the result.

2) **For each specimen, the interpretation of the results is as follows:**

A. When there are no missing melting peaks (the total number of melting peaks is more than or equal to 36):

a. When there are three wild peaks in each of the 12 channels of reaction A, B, and C without any mutant peak, it is determined as wild type (sensitive to pyrazinamide, Figure S2).

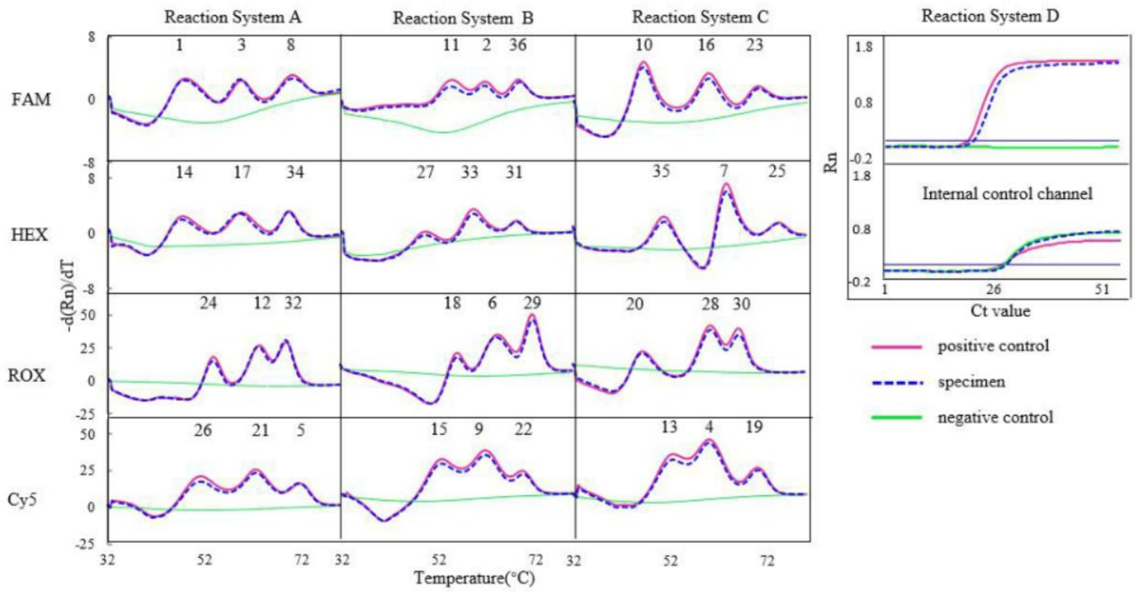

Figure S2 Typical result graph of wild-type

b. When there are at least three melting peaks in each of the twelve channels of reaction A, B, and C, and there are one or more mutant peaks, the specimen is determined as mutant type and is resistant to pyrazinamide (Figure S3).

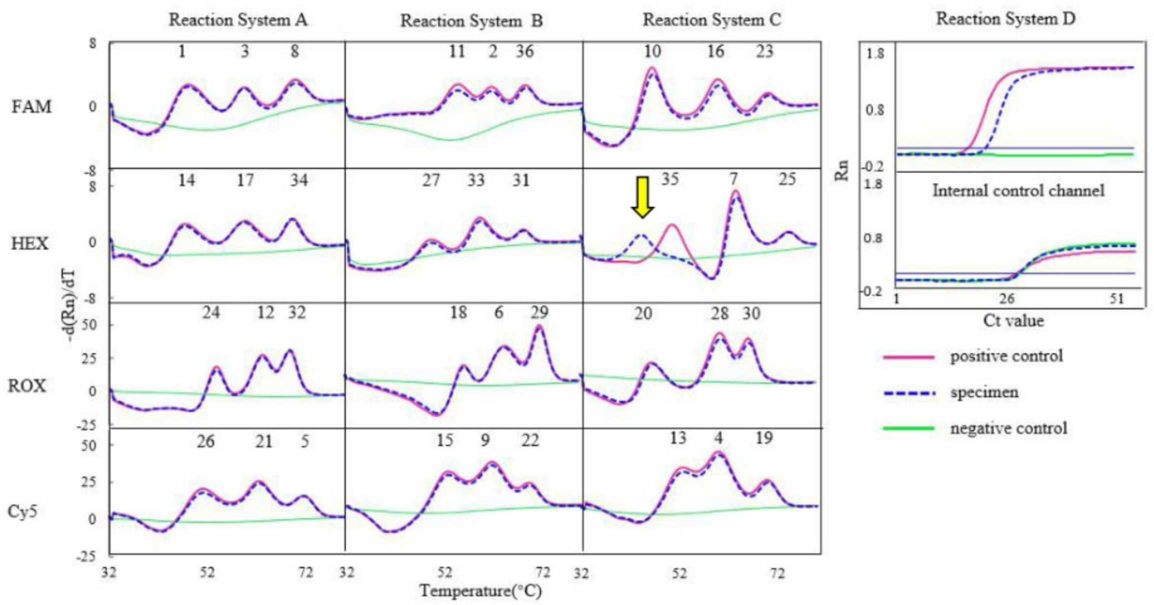

Figure S3 Typical result graph of PZA mutant type with mutant peak

B. When there are missing melting peaks (the total number of melting peaks is less than 36):

a. When the Ct <25.2 in the D-FAM channel and the Ct <27.0 in the D-HEX channel:

① If one or two melting peaks are missing in the 12 channels of reaction A, B, or C, the test sample is resistant to pyrazinamide (Figure S4);

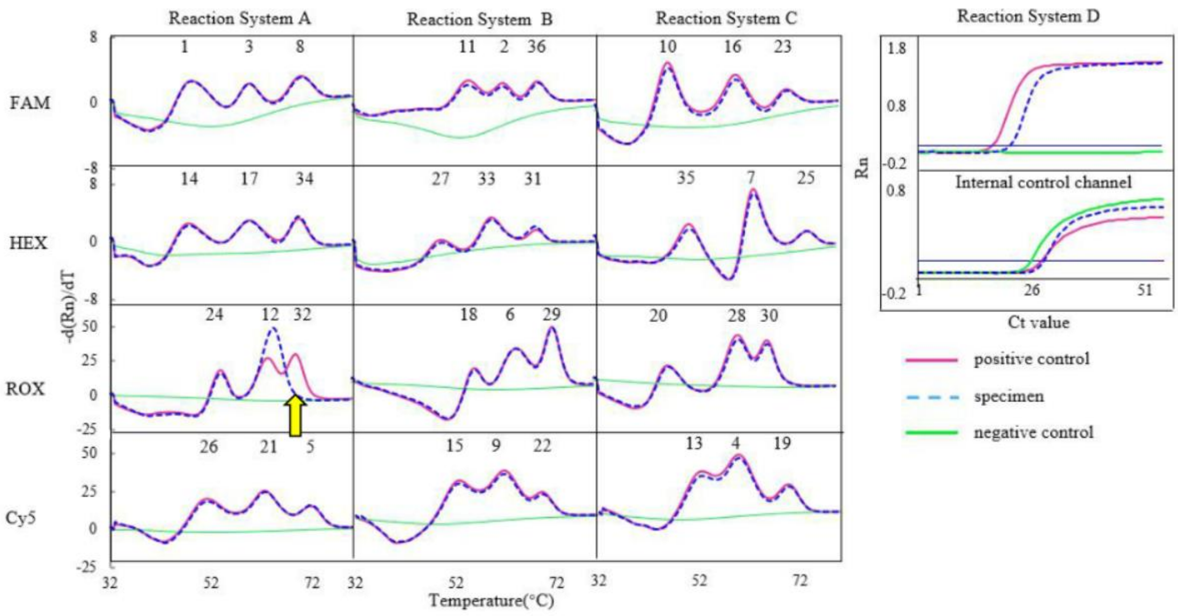

Figure S4 Typical result graph of PZA mutant type with missing mutant peak

② If three or more melting peaks are missing in the 12 channels of reaction A, B, or C, and the sequence number of missing melting peaks is continuous, there may be a short deletion in the target sequence, which means that the sample is resistant to pyrazinamide; if the missing melting peaks is discontinuous, the resistance of

this sample to pyrazinamide is unconfirmed.

b. When the Ct≥25.2 in the D-FAM channel and the Ct<27.0 in the D-HEX channel, it indicates that the sample concentration is too low to be detected, and the resistance to pyrazinamide is unconfirmed. A repeat sample extraction is recommended to confirm the result.

c. When the Ct≥27.0 or no signal in the D-HEX channel, it is indicated that the sample may contain a PCR inhibitor. Purification or appropriate dilution (e.g., 5 times dilution) of the template is recommended in this situation.

C. Invalid result: except for the above cases, a result may be invalid if no melting peak is shown in one or more channels. The possible reasons are: (a.) No or low MTB in the samples; (b.) Wrong operation; (c.) The kit is ineffective.

Warning: Environmental pollution in the laboratory, reagent contamination, and cross-contamination of specimens will lead to false-positive; improper transportation and/or storage, and incorrect reagent preparation may lower the accuracy of the test and lead to a false negative or inaccurate detection results.
